# Association of T-Cell Immunoglobulin and Mucin Domain-Containing Molecule 3 (Tim-3) Polymorphisms with Susceptibility and Disease Progression of HBV Infection

**DOI:** 10.1371/journal.pone.0098280

**Published:** 2014-05-27

**Authors:** Jingyu Liao, Qi Zhang, Yun Liao, Bei Cai, Jie Chen, Lixin Li, Lanlan Wang

**Affiliations:** Department of Laboratory Medicine, West China Hospital, Sichuan University, Chengdu, Sichuan, China; CRCL-INSERM, France

## Abstract

**Purpose:**

T-cell immunoglobulin and mucin domain-containing molecule 3 (Tim-3) plays an important role in regulating T cells in hepatitis B virus (HBV) infection and hepatocellular carcinoma (HCC). However, few researches have reported the association of Tim-3 genetic variants with susceptibility and progression of HBV infection. In this study, we focused on the association of Tim-3 polymorphisms with HBV infection, HBsAg seroclearance and hepatocellular carcinoma.

**Methods:**

A total of 800 subjects were involved in this study. Four groups were studied here, including HBV, HBsAg seroclearance, HBV-associated HCC and healthy controls. Three single-nucleotide polymorphisms (SNPs) of Tim-3, rs246871, rs25855 and rs31223 were genotyped to analyze the association of Tim-3 polymorphisms with susceptibility and disease progression of HBV infection.

**Results:**

Our study found that rs31223 and rs246871 were associated with disease progression of HBV infection, while none of the three SNPs was relevant to HBV susceptibility. The minor allele “C” of rs31223 was found to be associated with an increased probability of HBsAg seroclearance (P = 0.033) and genotype “CC” of rs246871 to be associated with an increased probability of HBV-associated HCC (P = 0.007). In accordance, haplotypic analysis of the three polymorphisms also showed that the haplotype block CGC* and TGC* were significantly associated with HBsAg seroclearance (P<0.05) while haplotype block CAT*, CGT*, TAC* and TGT* were significantly associated with HBV-associated HCC (all P<0.05).

**Conclusions:**

Genetic variants of Tim-3 have an important impact on disease progression of HBV infection. With specific Tim-3 polymorphisms, patients infected with HBV could be potential candidates of HCC and HBsAg seroclearance.

## Introduction

More than 240 million people worldwide are chronically infected with hepatitis B virus (HBV), causing 600 thousand people dead of hepatitis every year according to WHO's report in 2013. HBV persistency may lead to liver cirrhosis, liver failure, and hepatocellular carcinoma (HCC) which is the 6th most common neoplasm and the 3rd most frequent cause of cancer death [1], [2]. Previous studies have demonstrated that negative regulatory molecules including cytotoxic T lymphocyte antigen-4 (CTLA-4), B and T lymphocyte attenuator (BTLA), and programmed death-1 (PD-1) play important roles in antivirus and antitumor immunity [3], [4], [5], [6]. Accordingly, targeting negative regulatory molecules to restore proliferative ability and effector function of T cells has been regarded as a new treatment of virus infection and tumor [7].

T-cell immunoglobulin and mucin domain-containing molecule 3 (Tim-3) was recognized as a negative regulatory molecule, which was originally found to be expressed on Th1 cells and Tc1 cells [8], though recent studies suggested Tim-3 to be a potential marker for regulatory T cells [9], [10]. Interaction between Tim-3 and its ligand galectin-9 could induce apoptosis of Tim-3^+^Th1 cells [11], [12] and promote immunological tolerance [13], [14]. Studies on human immunodeficiency virus (HIV), Multiple Sclerosis, Chronic Hepatitis B (CHB) and Hepatitis C virus (HCV) infection have demonstrated an upregulation of Tim-3 on exhausted T cells, and blockade of Tim-3 signaling could restore T cell proliferation and cytokine production [15], [16], [17], [18], [19]. A study on HBV implied that Tim-3 might act as a potent regulator of antiviral T-cell responses [20]. Increased expression of Tim-3 on NK cells and T cell was found in CHB patients [21] and demonstrated to be associated with progression of CHB [22]. Blockade of Tim-3 signaling could restore anti-virus ability in CHB patients [17]. Since Tim-3 played an important role in HBV infection, researchers had further demonstrated that Tim-3^+^T cells were increased in patients with HBV-associated HCC and other cancers [9], [10], [23]. Blocking Tim-3 signaling could restore anti-tumor immunity, while high expression of Tim-3 on CD4^+^T cells was associated with greater metastatic risk or advanced cancer grades [23], [24]. Since there is a possibility of blocking Tim-3 signaling to enhance antivirus and antitumor immunity, researchers have combined anti–Tim-3 and anti–PD-L1 treatment in patients which resulted in a dramatic reduction in tumor growth while restored strong antivirus immunity [4], [25], [26], [27]. However, another study indicated that galectin-9 might enhance antitumor immunity via Tim-3 interactions [28]. Contradictory conclusions may be caused by different function of Tim-3 on innate immune cells and adaptive immune cells that Tim-3 transmitted negative signals to T cells while playing a positive role on NK cells [29], [30].

Previous studies have implied the association of Tim-3 with HBV infection and HBV-associated HCC [16], [17], [22], [23]. However, the expression and function of Tim-3 in HBsAg seroclearance still remains unclear. Considering the genetic variants of Tim-3 might influence its expression and further impact the function when infected with HBV, thus we designed a retrospective case-control study to assess the association of Tim-3 polymorphisms with the susceptibility and disease progression of HBV infection.

## Materials and Methods

### Ethics Statement

This study was approved by the ethics committee of West China Hospital of Sichuan University, performed after obtaining written informed consents from all subjects and carried out in accordance with the guidelines of the 1975 Declaration of Helsinki.

### Patients

800 subjects were recruited in this study meeting the following inclusion criteria: (1) HBV patients with positive HBsAg. (2) Patients with HBsAg seroclearance, who were detected loss of serum HBsAg with or without appearance of anti-HBs. These patients were selected based on serology test results in retrospect. (3) Patients with HBV-associated hepatocellular carcinoma, confirmed by histopathology, CT or MRI and serology test. (4) Healthy controls, without any history of hepatitis or other liver diseases with healthy liver function. Exclusion criteria: (1) Patients co-infected with HCV, HIV or other virus were excluded. (2) Patients with autoimmune diseases including autoimmune hepatitis, systemic lupus erythematosus, rheumatoid arthritis, sicca syndrome and other autoimmune diseases were excluded. (3) Patients with other tumor diseases were excluded. Here, we should mention that patients diagnosed as HCC with cirrhosis were not excluded, because no significant differences were found in Tim-3 polymorphisms distribution as well as tumor stage between HCC patients and HCC with cirrhosis patients (Table S1 and Table S2).

### Selection of Tim-3 SNPs and Genotyping

We selected three representative Tim-3 SNPs which had the minor allele frequency of >10% in Chinese Han population according to the International HapMap Project (www.hapmap.org). Three SNPs studied here were rs246871, rs31223 and rs25855 chosen by Haploview software (version 4.2; Daly Lab at the Broad Institute, Cambridge, MA 02141, USA). Rs246871 (156585254T>C), rs25855 (156624400A>G, in the intron region) and rs31223 (156625279T>C, in the intron region) were selected because their MAFs are higher than other SNPs according to Haploview software. SNPs were genotyped using high resolution melt (HRM) - Real-time PCR analysis in Light Cycler 480 System (Roche Applied Science, Germany). Primers were designed using Primer Premier 5.0 software and synthesized by Bibliog Technologies LTD (Shanghai, China) (Table S3).

### DNA Extraction and HRM-PCR Reaction Condition

Genomic DNA was extracted from peripheral blood samples using BioTeke DNA Blood kit according to the manufacturer's instructions and was stored at −80°C.

PCR components included Master Mix (520 ul; Roche) containing FastStart Taq DNA polymerase reaction buffer, dNTPs and HRM dye; 10.4 ul each forward and reverse primers of rs246871/rs31223/rs25855 respectively; 25 nM MgCl_2_ (Roche). Wild-type and mutant homozygotes were distinguished by spiking samples with a known genotype obtained by sequencing previously before PCR. Unknown genomic DNA (10 ng) was used as template. H_2_O (Roche) was added to bring the final reaction volumes to 1040 ul.

PCRs were conducted in 96-well plates in 10 ul-volumes using the following touchdown PCR cycling and HRM conditions: initiation with a 10-min hold at 95°C, 46 cycles of 95°C for 15 s, touchdown cycling (decreasing 1°C/cycle), annealing in the range of 65–55°C for 10 s, and 72°C for 20 s. Following amplification, samples were heated to 95°C for 1 min and then cooled to 40°C for 1 min encouraging heteroduplex formation. HRM curve data were obtained by melting over the range 65–95°C at a rate of 15 data acquisitions per 1°C. Results were analyzed using Light Cycler 480 Gene Scanning software (Roche Applied Science, Germany).

### Statistical Analysis

Continuous variables following normal distribution were expressed in mean ± STD (range), while those with abnormal distribution expressed in median (range). The Student't test or Mann-Whitney U test, or the Kruskal-Wallis H test when appropriate, was used for continuous variables with a skewed distribution. The Pearson χ^2^ test, or Fisher exact test when appropriate, was used for categorical variables. Confounding factors and odds ratios were adjusted for age and sex by using multi-factor Logistic regression analysis.

Hardy-Weinberg equilibrium for the genotypic distribution of Tim-3 polymorphisms in all patient groups was determined. When comparing patient groups, the following association analytical methods were used: comparing allelic frequencies (major allele “A” vs. minor allele “B”), dominant gene model (AA vs. AB + BB), recessive gene model (AA+AB vs. BB) and additive gene model (AA vs. AB vs. BB).

Haplotype analysis was performed by analyzing the 3 polymorphisms of Tim-3 as haplotype blocks. Compared to single SNP association analysis, haplotype-based association analysis was more sensitive [31] which could capture additional phenotype-related variants. All statistical analyses were performed using SPSS version 17.0 (SPSS Inc, Chicago, Illinois) and Shesis online software. A 2-tailed P value <0.05 was considered statistically significant.

## Results

### Patients

The clinical characteristics of the four groups were depicted in Table 1. Patients' age and sex were not completely matched between groups. There were significant differences in the distribution of age among HBV, HBsAg Seroclearance and HBV-associated HCC (all P<0.05). In addition, the distribution of sex had no significant differences only between HBV and HBsAg seroclearance (P>0.05). Besides, liver biochemistry indexes among the four groups were not matched, either (all P<0.05). However, considering that genetic variants were relevant to host factors, such as age and sex instead of biochemistry indexes, thus age and sex were adjusted in following comparison when analyzing the association of Tim-3 polymorphisms with HBV, HBsAg seroclearance and HBV-associated HCC.

**Table 1:** Clinical Demographics of the Four Patient Groups.

Demographic	Healthy controls	Patients with HBV	Patients with HBsAg Seroclearance	Patients with HBV-associated HCC	P_1_ value a	P_2_ value b	P_3_ value c
No. of patients	200	200	200	200			
Age, y	43.04±14.54 (18.00–69.00)	44.74±13.15 (18.00–69.00)	51.57±13.29 (23.00–83.00)	52.22±11.62 (19.00–82.00)	0.199	<0.001	<0.001
Sex (male %)	87(44.0%)	127(63.5%)	109(54.5%)	150(75.0%)	<0.001	0.084	0.017
Albumin, g/dL	42.98(3.1–54.9)	45.10(20.5–52.1)	45.2(21.7–54.4)	41.35(21.3–49.9)	0.048	0.055	<0.001
ALT, U/L	18.00(4–186)	28.00(6–1508)	21.00(6–99)	40.00(7–3513)	<0.001	<0.001	<0.001
AST, U/L	20.00(6–235)	26.5(7–882)	22.00(12–119)	40.50(4–456)	<0.001	<0.001	<0.001

a P_1_ value was calculated between HBV and healthy controls.

b P_2_ value was calculated between HBV and HBsAg Seroclearance.

c P_3_ value was calculated between HBV and HCC. P<0.05 was considered statistically significant.

Abbreviations: HBV, Hepatitis B virus; HCC, Hepatocellular Carcinoma; ALT, alanine aminotransferase; AST, aspartate aminotransferase.

### Genotypic Distribution of the Three SNPs

The genotypic distribution of the 3 polymorphisms was depicted in Table 2. The minor allelic frequencies (MAFs) of each SNP were as follows: rs246871, 0.384; rs25855, 0.198; rs31223, 0.395. The Hardy-Weinberg calculations for all 3 polymorphisms were shown in Table 3. All of the three polymorphisms in healthy controls were in Hardy-Weinberg equilibrium (all P>0.05).

**Table 2:** Distribution of Tim-3 Polymorphisms in All Groups.

SNP	Genotype	HBV	HBsAg Seroclearance	HBV-associated HCC	Healthy Control
rs246871	TT	120(60.00%)	117(58.50%)	115(57.50%)	111(55.50%)
(T>C)	CT	69(34.50%)	76(38.00%)	58(29.00%)	73(36.50%)
	CC	11(5.50%)	7(3.50%)	27(13.50%)	16(8.00%)
Allele	T	309(77.25%)	310(77.50%)	288(72.00%)	295(73.75%)
	C	91(22.75%)	90(22.50%)	112(28.00%)	105(26.25%)
rs25855	GG	68(34.00%)	90(45.00%)	90(45.00%)	74(37.00%)
(G>A)	AG	105(52.50%)	83(41.50%)	84(42.00%)	102(51.00%)
	AA	27(13.50%)	27(13.50%)	26(13.00%)	24(12.00%)
Allele	G	241(60.00%)	263(66.00%)	264(66.00%)	250(63.00%)
	A	159(40.00%)	137(34.00%)	136(34.00%)	150(38.00%)
rs31223	TT	71(35.50%)	64(32.00%)	71(35.50%)	80(40.00%)
(T>C)	CT	98(49.00%)	82(41.00%)	95(47.50%)	90(45.00%)
	CC	31(15.50%)	54(27.00%)	34(17.00%)	30(15.00%)
Allele	T	240(60.00%)	210(52.50%)	237(59.25%)	250(62.50%)
	C	160(40.00%)	190(47.50%)	163(40.75%)	150(37.50%)

Distribution of each SNP was shown in number and frequency.

**Table 3:** Hardy-Weinberg Calculations for All Three Polymorphisms in All Groups.

SNP	Patient Group	χ^2^	P value
rs246871	HBV infection	0.657	0.418
	HBsAg seroclearance	1.606	0.205
	HBV-associated HCC	15.765	<0.001
	Controls	0.321	0.571
rs25855	HBV infection	1.846	0.174
	HBsAg seroclearance	1.652	0.199
	HBV-associated HCC	0.824	0.364
	Controls	1.549	0.213
rs31223	HBV infection	0.087	0.768
	HBsAg seroclearance	6.333	0.012
	HBV-associated HCC	0.053	0.817
	Controls	0.320	0.572

Genotypic distribution was considered in Hardy-Weinberg equilibrium when P>0.05

### Association of Tim-3 Polymorphisms with HBV Infection, HBsAg Seroclearance and HBV-Associated HCC

The association analysis of the three SNPs with HBV, HBsAg seroclearance and HBV-associated HCC was shown in Table 4. All three SNPs had no association with HBV susceptibility (all P>0.05). However, the minor allele “C” of rs31223 was associated with an increased probability of HBsAg seroclearance [allelic frequency model: P = 0.033, odds ratio (OR) = 1.359, 95% confidence interval (CI) = 1.026–1.801; recessive gene model: P = 0.003, OR = 2.170, 95%CI = 1.302–3.615; additive gene model: P = 0.012], while rs25855 was associated with a decreased probability of HBsAg seroclearance (dominant gene model: P = 0.037, OR = 0.640, 95%CI = 0.422–0.973). The remaining SNP rs246871 had no association with HBsAg seroclearance (all P>0.05), but was relevant to HBV-associated HCC (recessive gene model: P = 0.007, OR = 2.781, 95%CI = 1.329–5.820; additive gene model: P = 0.023). On the contrary, SNP rs25855 was associated with a reduced probability of HBV-associated HCC (dominant gene model: P = 0.031, OR = 0.625, 95%CI = 0.409–0.957). The remaining SNP rs31223 had no association with HBV-associated HCC (all P>0.05). All these results were adjusted for age and sex.

**Table 4:** Association of Tim-3 Polymorphisms with Susceptibility of HBV Infection, HBsAg Seroclearance and HBV-Associated HCC.

SNP	Test^a^	P_adj_*(OR, 95%CI)^b^	P_adj_**(OR, 95%CI)^c^	P_adj_***(OR, 95%CI)^d^
rs246871	Allelic	0.277(0.831,0.596–1.160)	0.936(0.986,0.708–1.375)	0.137(1.276,0.925–1.760)
(T>C)	Dominant	0.407(0.842,0.562–1.263)	0.717(1.079,0.714–1.631)	0.444(1.179,0.774–1.795)
	Recessive	0.472(0.748,0.339–1.650)	0.314(0.605,0.227–1.611)	0.007(2.781,1.329–5.820)
	Additive	0.632	0.484	0.023
	TT	reference	reference	reference
	CT	0.528(0.871,0.568–1.336)	0.507(1.156,0.753–1.777)	0.702(0.914,0.577–1.449)
	CC	0.405(0.710,0.316–1.592)	0.376(0.639,0.237–1.723)	0.010(2.696,1.267–5.738)
rs25855	Allelic	0.797(1.039,0.776–1.391)	0.247(0.843,0.632–1.125)	0.087(0.776,0.581–1.037)
(G>A)	Dominant	0.700(1.086,0.714–1.651)	0.037(0.640,0.422–0.973)	0.031(0.625,0.409–0.957)
	Recessive	0.770(1.094,0.600–1.994)	0.726(1.110,0.619–1.992)	0.967(1.013,0.548–1.873)
	Additive	0.912	0.063	0.072
	GG	reference	reference	reference
	AG	0.753(1.073,0.693–1.660)	0.020(0.591,0.380–0.921)	0.022(0.591,0.377–0.927)
	AA	0.694(1.140,0.593–2.195)	0.575(0.834,0.443–1.572)	0.424(0.764,0.394–1.480)
rs31223	Allelic	0.514(1.102,0.824–1.473)	0.033(1.359,1.026–1.801)	0.817(1.034,0.778–1.375)
(T>C)	Dominant	0.361(1.213,0.802–1.833)	0.335 (1.235,0.804–1.897)	0.996(1.001,0.650–1.542)
	Recessive	0.770(1.087,0.623–1.897)	0.003(2.170,1.302–3.615)	0.646(1.140,0.652–1.991)
	Additive	0.659	0.012	0.889
	TT	reference	reference	reference
	CT	0.386(1.213,0.783–1.880)	0.865(0.961,0.604–1.527)	0.875(0.964,0.610–1.524)
	CC	0.539(1.210,0.659–2.222)	0.010(2.120,1.193–3.768)	0.727(1.116,0.603–2.067)

a Allelic: major allele “A” vs. minor allele “B”; dominant gene mode: AA vs. AB + BB; recessive gene mode: AA + AB vs. BB. Additive gene mode: AA vs. AB vs. BB.

b P* value was calculated between HBV and healthy controls.

c P** value was calculated between HBV and HBsAg Seroclearance.

d P*** value was calculated between HBV and HCC. All results were adjusted for age and sex. P<0.05 was considered statistically significant.

Abbreviations: SNP, single-nucleotide polymorphism; CI, confidence interval; OR, odds ratio.

### Haplotype Analysis of Tim-3 Polymorphisms with HBV infection, HBsAg Seroclearance and HBV-Associated HCC

Haplotypes were constructed based on the three SNPs. Results were depicted in Table 5. Based on the Tim-3 haplotype constructed from SNPs rs246871 (minor allele “C”), rs25855 (minor allele “A”), and rs31223 (minor allele “C”), when comparing the target haplotype with remaining haplotypic combinations, haplotype block CAT* and CGT* were significantly associated with HBV infection (CAT*: P<0.001, OR = 2139.7, 95%CI = 132.85–34463.64; CGT*: P = 0.006, OR = 0.584, 95%CI = 0.398–0.857). All other haplotypic combinations showed no significance with HBV infection. Haplotype block CGC* and TGC* were significantly associated with HBsAg seroclearance (CGC*: P = 0.040, OR = 1.997, 95%CI = 1.022–3.902; TGC*: P = 0.043, OR = 1.358, 95%CI = 1.010–1.826). All other haplotypic combinations showed no significance with HBsAg seroclearance. Haplotype block CAT*, CGT*, TAC* and TGT* were significantly associated with HBV-associated HCC (CAT*: P<0.001, OR = 0.107, 95%CI =  0.028–0.412; CGT*: P = 0.002, OR = 1.825, 95%CI = 1.248–2.669; TAC*: P = 0.013, OR = 0.368, 95%CI = 0.162–0.836; TGT*: P = 0.003, OR = 0.508, 95%CI = 0.321–0.805). All other haplotypic combinations showed no significance with HBV-associated HCC.

**Table 5:** Haplotype Analysis of Tim-3 Polymorphisms with HBV Infection, HBsAg Seroclearance and HBV-Associated HCC.

	HBV Infection(freq)	HBsAg Seroclearance (freq)	HBV-Associated HCC (freq)	Healthy Control (freq)	P*(OR, 95%CI)^a^	P**(OR, 95%CI)^b^	P***(OR, 95%CI)^c^
CAT*^d^	21.16(0.053)	20.57(0.051)	2.37(0.006)	0.01(0.000)	<0.001 (2139.7,132.85∼34463.6)	0.907 (0.963,0.516∼1.797)	<0.001(0.107,0.028∼0.412)
CGC*	13.59(0.034)	26.41(0.066)	19.06(0.048)	17.22(0.043)	0.496(0.778,0.376∼1.606)	0.040(1.997,1.022∼3.902)	0.327(1.425,0.700∼2.899)
CGT*	51.05(0.128)	39.55(0.099)	84.11(0.210)	79.65(0.199)	0.006(0.584,0.398∼0.857)	0.187(0.744,0.479∼1.156)	0.002(1.825,1.248∼2.669)
TAC*	21.36(0.053)	13.45(0.034)	8.13(0.020)	10.80(0.027)	0.059(2.023,0.960∼4.264)	0.164(0.612,0.305∼1.229)	0.013(0.368,0.162∼0.836)
TAT*	110.28(0.276)	103.52(0.259)	119.04(0.298)	131.06(0.328)	0.098(0.774,0.571∼1.049)	0.548(0.908,0.663∼1.243)	0.488(1.115,0.820∼1.517)
TGC*	118.85(0.297)	146.67(0.367)	129.35(0.323)	113.86(0.285)	0.729(1.056,0.777∼1.434)	0.043(1.358,1.010∼1.826)	0.417(1.133,0.838∼1.530)
TGT*	57.51(0.144)	46.36(0.116)	31.48(0.079)	39.28(0.098)	0.051(1.535,0.996∼2.365)	0.226(0.774,0.511∼1.172)	0.003(0.508,0.321∼0.805)

a P* value was calculated between healthy controls and HBV Infection.

b P** value was calculated between HBV Infection and HBsAg Seroclearance.

c P*** value was calculated between HBV Infection and HBV-Associated HCC. P<0.05 was considered statistically significant.

d Order of haplotype block: rs246871 (minor allele “C”), rs25855 (minor allele “A”), rs31223 (minor allele “C”).

## Discussion

Our study demonstrated that Tim-3 polymorphisms were associated with HBsAg seroclearance and HBV-associated HCC, illustrating the importance of host factors in influencing HBsAg seroclearance and HCC carcinogenesis. To our knowledge, the present study is the first to determine the role of Tim-3 polymorphisms with HBV disease progression.

The involvement of Tim-3 polymorphisms is an evident at the level of gene expression. The positive association of Tim-3 haplotype CGC* and TGC* as well as genotype “CC” of rs31223 with HBsAg seroclearance (Table 4, Table 5) is an intriguing finding, which implies that “C” of rs31233 is a protective allele when infected with HBV. Since the influence of Tim-3 on HBsAg seroclearance remains unknown, more researches are needed to further confirm this association.

Previous studies have demonstrated the association of Tim-3 with HBV infection and HBV-associated HCC, that blockade of Tim-3 signaling could restore antivirus as well as antitumor immunity [16], [17], [22], [23], suggesting an important role of Tim-3 in HBV infection and HBV-associated HCC. In our study, we found genotype “CC” and haplotype block CGT* of rs246871 to be associated with an increased probability of HBV-associated HCC, while TAC* and TGT* were associated with a decreased probability (Table 5), indicating the allele “C” of rs246871 might increase the risk of hepatocarcinogenesis in HBV infected patients. However, haplotype block CAT* was found with a decreased probability of HBV-associated HCC (P<0.001, OR = 0.107). This contradictory result might be caused by an interaction between allele “C” of rs246871 and allele “A” of rs25855, that the allele “A” was found no association but a tendency with HBV-associated HCC (dominant model: P = 0.031; OR = 0.625). Though all three SNPs were found no association with susceptibility to HBV infection (all P>0.05), it should be notable that haplotype block CGT* was significantly associated with a decreased probability of HBV, implying this haplotype of Tim-3 might reduce the risk of HBV infection.

Our findings suggested a possibility of Tim-3 polymorphisms to enhance or inhibit the host immune response when infected with hepatitis B virus. Tim-3 polymorphisms could have an impact on the effector function of T cells, thus further influence the disease progression of HBV infection. Though no significance was found in susceptibility to HBV infection, genetic variants of Tim-3 was suggested to influence the disease progression of HBV patients in our study. Patients with favorable Tim-3 polymorphisms could potentially develop HBV-associated hepatocellular carcinoma or HBsAg seroclearance.

There are several limitations of this study. First, this study was lack of data on HBV genotyping. Though Chinese patient cohort would likely be either genotype B or C, the influence of HBV genotype should still be further studied. Second, cases and controls were not matched for age and sex, but both of the two factors were adjusted by multi-factor regression in our study. Third, the expression of Tim-3 and function of Tim-3^+^T cells were not detected in any group. Future function and expression experiments would be needed to address the effect of these SNPs in hepatocarcinogenesis and HBsAg seroclearance.

## Conclusion

Tim-3 polymorphisms could influence the disease progression of HBV infection, while no association was found with HBV susceptibility. With special Tim-3 polymorphisms, patients could be potential candidates of HBV-associated HCC or HBsAg seroclearance, as illustrated by gene type and haplotype analysis of Tim-3 polymorphisms. Further studies would be aimed at functional and expressional studies of Tim-3, in order to validate the effect of these SNPs in hepatocarcinogenesis and seroclearance.

## Supporting Information

Table S1 **Characteristics of Patients with HCC.**
(DOC)

Table S2 **Distribution of Tim-3 Polymorphisms in Patients with or without Liver Cirrhosis.**
(DOC)

Table S3 **PCR Primers and Amplicons.**
(DOC)
