# Supplementary material for: Association of T-Cell Immunoglobulin and Mucin Domain-Containing Molecule 3 (Tim-3) Polymorphisms with Susceptibility and Disease Progression of HBV Infection
Source: PLoS One. 2014 May 27;9(5):e98280. doi: 10.1371/journal.pone.0098280 (PMC4035322; doi:10.1371/journal.pone.0098280)
Supplement: Table S1 — Characteristics of Patients with HCC. (DOC) [file pone.0098280.s001.doc]

Table S1. Characteristics of Patients with HCC

| Demographic | HCC without cirrhosis | HCC with cirrhosis | *P* value |
| --- | --- | --- | --- |
| No. of patients | 83 | 117 |  |
| Age, y | 51.81±12.37 | 52.50±11.092 | 0.677 |
| Sex (male %) | 53(63.9%) | 97(82.9%) | 0.002 |
| Albumin, g/dL | 41.171±4.966 | 40.27±5.265 | 0.222 |
| ALT, U/L | 31.00 | 46.00 | 0.002 |
| AST, U/L | 34.00 | 46.00 | 0.002 |
| PT, s | 11.792±1.015 | 12.403±1.294 | 0.001 |
| INR | 1.062±0.096 | 1.120±0.118 | 0.001 |
| Tumor stage |  |  | 0.128 |
| Stage 0 | 11(13.3%) | 6(5.1%) |  |
| Stage I | 12(14.5%) | 23(19.7%) |  |
| Stage II | 14(16.9%) | 31(26.5%) |  |
| Stage III | 23(27.7%) | 31(26.5%) |  |
| Stage IV | 23(27.2%) | 26(22.2%) |  |

P value was calculated between HCC without cirrhosis and HCC with cirrhosis.

P value < 0.05 was considered statistically significant.

Abbreviations: HCC, Hepatocellular Carcinoma; ALT, alanine aminotransferase; AST, aspartate aminotransferase; PT, prothrombin time; INR, international normalized ratio.
