# Supplementary material for: Association of T-Cell Immunoglobulin and Mucin Domain-Containing Molecule 3 (Tim-3) Polymorphisms with Susceptibility and Disease Progression of HBV Infection
Source: PLoS One. 2014 May 27;9(5):e98280. doi: 10.1371/journal.pone.0098280 (PMC4035322; doi:10.1371/journal.pone.0098280)
Supplement: Table S2 — Distribution of Tim-3 Polymorphisms in Patients with or without Liver Cirrhosis. (DOC) [file pone.0098280.s002.doc]

Table S2. Distribution of Tim-3 Polymorphisms in Patients with or without Liver Cirrhosis

| SNP | Genotype | HCC without cirrhosis (n=83) | HCC with cirrhosis (n=117) | *P* value |
| --- | --- | --- | --- | --- |
| rs246871 | TT | 49(59.0%) | 66(56.4%) | 0.679 |
| （T>C） | CT | 25(30.1%) | 33(28.2%) |  |
|  | CC | 9(10.8%) | 18(15.4%) |  |
| Allele | T | 123(74.1%) | 165(70.5%) | 0.498 |
|  | C | 43(25.9%) | 69(29.5%) |  |
| rs25855 | GG | 35(42.2%) | 55(47.0%) | 0.378 |
| （G>A） | AG | 34(41.0%) | 50(42.7%) |  |
|  | AA | 14(16.9%) | 12(10.3%) |  |
| Allele | G | 104(62.7%) | 160(68.4%) | 0.241 |
|  | A | 62(37.3%) | 74(31.6%) |  |
| rs31223 | TT | 30(36.1%) | 41(35.0%) | 0.682 |
| （T>C） | CT | 37(44.6%) | 58(49.6%) |  |
|  | CC | 16(19.3%) | 18(15.4%) |  |
| Allele | T | 97(58.4%) | 140(59.8%) | 0.836 |
|  | C | 69(41.6%) | 94(40.2) |  |

P value: HCC without cirrhosis vs. HCC with cirrhosis.

P value < 0.05 was considered statistically significant.

Abbreviations: HCC, Hepatocellular Carcinoma; SNP, single-nucleotide polymorphism.
