# Supplementary material for: Association of T-Cell Immunoglobulin and Mucin Domain-Containing Molecule 3 (Tim-3) Polymorphisms with Susceptibility and Disease Progression of HBV Infection
Source: PLoS One. 2014 May 27;9(5):e98280. doi: 10.1371/journal.pone.0098280 (PMC4035322; doi:10.1371/journal.pone.0098280)
Supplement: Table S3 — PCR Primers and Amplicons. (DOC) [file pone.0098280.s003.doc]

Table S3. PCR Primers and Amplicons

| Polymorphism | Direction | Sequence | Amplicon size (bp) | Annealing Temperature(℃) |
| --- | --- | --- | --- | --- |
| rs246871 | Forward | CCTTCCTGGCTATCATCAACACTT | 128 | 61.7 |
|  | Reverse | CAAAACTCTGTCTCAAAATCCGCT |  | 62.1 |
| rs25855 | Forward | GCATACCCCTTATTCCTCTCCCA | 116 | 61.3 |
|  | Reverse | AGTGTGAAGCAGGAGGAAAAGCA |  | 62.6 |
| rs31223 | Forward | CCCACACAGGTATCTGCTCTTCAT | 177 | 62.3 |
|  | Reverse | GCCACCCAAATGAAAAAAGTGAGAC |  | 65.3 |
